# Genomic profiling guided therapy for synchronous SCC and CLL of the parotid gland: a rare case report

**DOI:** 10.1093/jscr/rjaf752

**Published:** 2025-09-24

**Authors:** Rockey Dahiya, Natalie Weiss, Prishae Wilson, Bastien A Valencia-Sanchez, Phillip Pirgousis

**Affiliations:** Department of Otolaryngology - Head & Neck Surgery, Mayo Clinic, 4500 San Pablo Rd S, Jacksonville, FL 32224, United States; Department of Otolaryngology - Head & Neck Surgery, Mayo Clinic, 4500 San Pablo Rd S, Jacksonville, FL 32224, United States; Department of Otolaryngology - Head & Neck Surgery, Mayo Clinic, 4500 San Pablo Rd S, Jacksonville, FL 32224, United States; Department of Otolaryngology - Head & Neck Surgery, Mayo Clinic, 4500 San Pablo Rd S, Jacksonville, FL 32224, United States; Department of Otolaryngology - Head & Neck Surgery, Mayo Clinic, 4500 San Pablo Rd S, Jacksonville, FL 32224, United States

**Keywords:** synchronous tumors, squamous cell carcinoma (SCC), chronic lymphocytic leukemia (CLL), genetic profiling, parotid gland, parotidectomy

## Abstract

Synchronous squamous cell carcinoma (SCC) and chronic lymphocytic leukemia (CLL) in the parotid gland is rare, with limited evidence on personalized treatment. We report a 75-year-old male with prior cutaneous SCC who presented with a hypermetabolic parotid mass and cervical lymphadenopathy; fine needle aspiration confirmed SCC. Surgery revealed poorly differentiated SCC and CLL in multiple lymph nodes. He underwent radiotherapy but developed regional SCC relapse without systemic CLL symptoms. Recurrence in the ear required extensive surgical resection and reconstruction. Genetic profiling showed high tumor mutational burden (>50 mutations/Mb) and mutations in ARID1B, CDKN2A, MSH2, PMS2, and TP53. He received six cycles of cemiplimab followed by cetuximab-based targeted therapy, based on rising circulating DNA levels. This case emphasizes the value of genetic profiling and tools like TMB, FISH, and immunohistochemistry for risk stratification and personalized treatment in managing advanced or complex parotid malignancies, including synchronous SCC and CLL, to optimize patient outcomes.

## Introduction

Synchronous tumors involve simultaneous, distinct primary tumors. In the parotid gland, they are rare, with a 3%–5% incidence, commonly involving pleomorphic adenoma and oncocytic carcinoma, Warthin’s tumor and mucoepidermoid carcinoma, and sebaceous lymphadenoma and membranous basal cell adenoma [1–4]. While chronic lymphocytic leukemia (CLL) and squamous cell carcinoma (SCC) have been reported, genetic profile-guided personalized treatment strategies remain underutilized. The American Society of Clinical Oncology states that, outside of specific scenarios where a particular diagnosis linked to a targetable alteration (e.g. androgen receptor in salivary duct carcinoma of ETV6-NTRK3 in secretory carcinoma), routine broad genomic profiling is not recommended for most salivary gland tumors [5]. However, in advanced or complex cases, including synchronous malignancies, genomic profiling may be considered for both diagnostic and investigational purposes [6].

We present a case of synchronous SCC and CLL and discuss implications for oncologic management and the role of genomic profiling. Informed consent was obtained from the patient for review of his case, and the electronic medical record was queried for regarding details diagnosis, management, and outcomes. This was reported per SCARE criteria for surgical case reports [6].

## Case report

**Figure 1:**
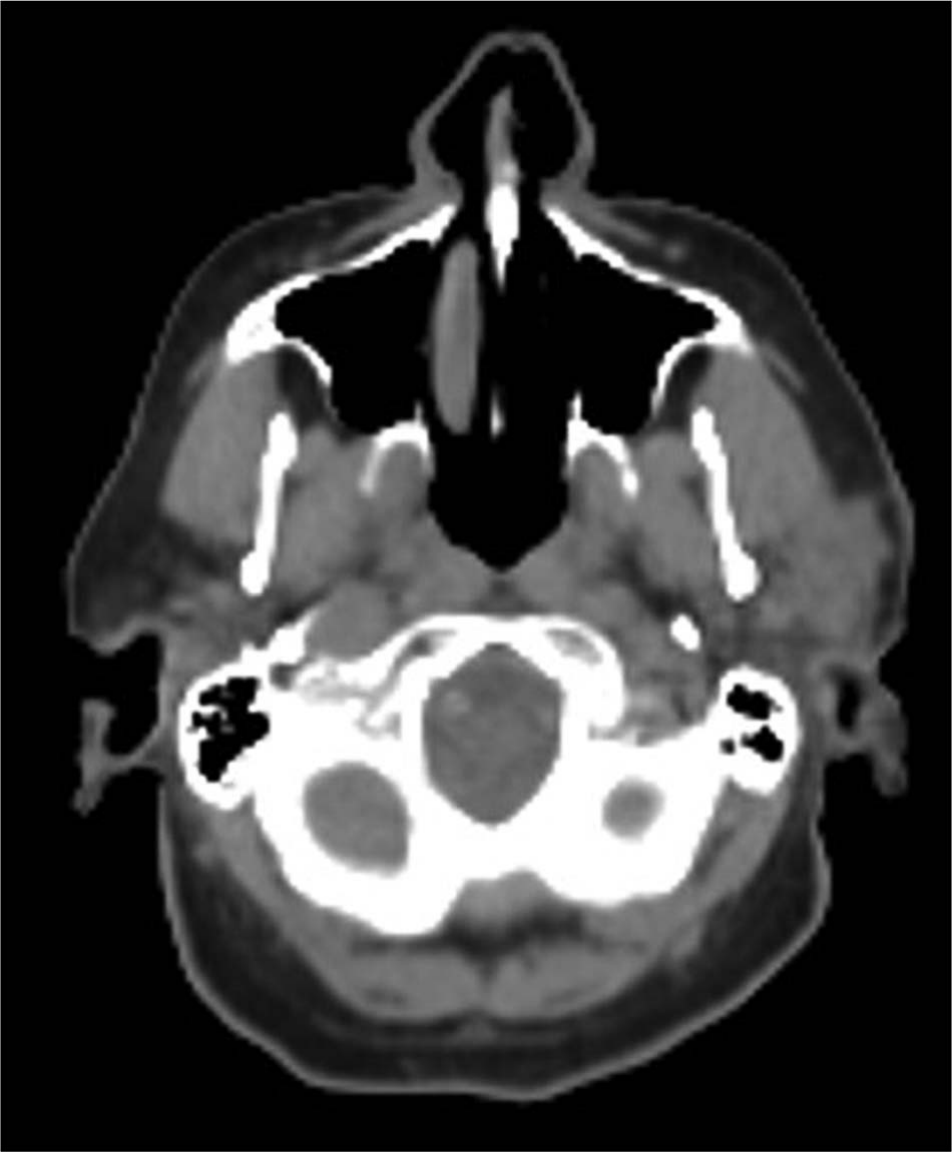
Hypermetabolic mass in the superficial anterior left parotid gland measuring 2.8 × 2.5 × 2.6 cm.

A 75-year-old male presented with several months of left preauricular and upper neck pain. His history included multiple basal and SCCs, Mohs surgery for SCC on the scalp vertex, and a family history of skin cancer. Neck ultrasound revealed anechoic complex areas near the left parotid gland. Imaging identified a hypermetabolic 2.6 × 1.9 × 2.5 cm mass in the superficial parotid and fluorodeoxyglucose (FDG)-avid bilateral lymphadenopathy in level I-III (Fig. 1). FNA confirmed SCC, positive for Keratin, p40, and p63, and negative for Melan A and SOX 10.

**Figure 2:**
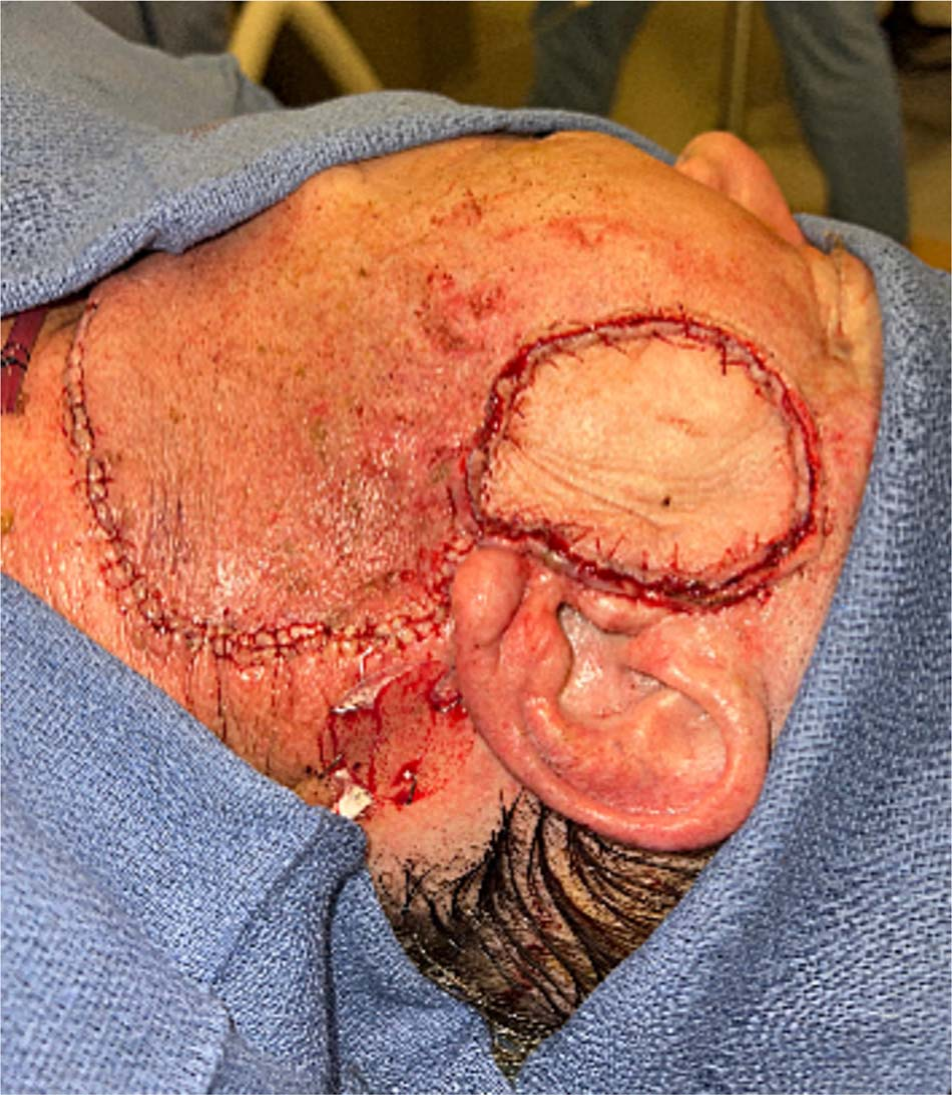
Parotid mass.

**Figure 3:**
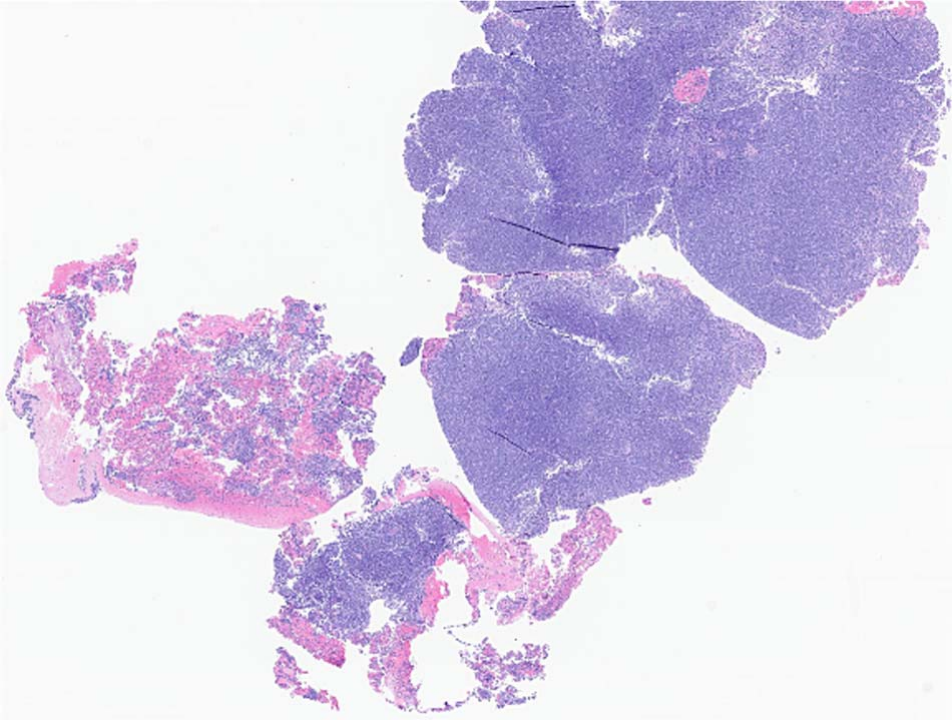
Left parotid tumor, biopsy: small lymphocytic lymphoma/CLL involving a lymph node.

The patient underwent wide local excision of preauricular skin, parotidectomy, and selective left neck dissection (levels I–IV) (Fig. *2*). Surgical pathology revealed poorly differentiated SCC involving subcutis, parotid, and surrounding soft tissues. In addition, CLL was found in three intra-parotid lymph nodes (Fig. 3). Immunohistochemistry showed cells positive for CD20, CD5, CD19, CD22, CD23, CD41, and BCL2, and negative for CD3, CD10, CD21, and cyclin D1. Flow cytometry revealed lambda light chain-restricted B-cells, with an absolute clonal B-cell count of 1.01 × 10^9^/l, and 16.7% atypical lymphocytes in peripheral blood.

The patient received five cycles of radiotherapy targeting high-risk areas. Immunotherapy was deferred due to absence of positive margins and metastatic disease in the cervical lymph nodes. Hematologic evaluation showed no systemic symptoms but mild thrombocytopenia and lymphocytosis. Six months later, he developed facial weakness, ear canal itching, a 3 cm ulcerated postauricular growth, and a small subcentimeter mastoid nodule. A positron emission tomography - computed tomography scan (PET-CT) demonstrated in-field SCC relapse. A biopsy confirmed invasive SCC recurrence.

**Figure 4:**
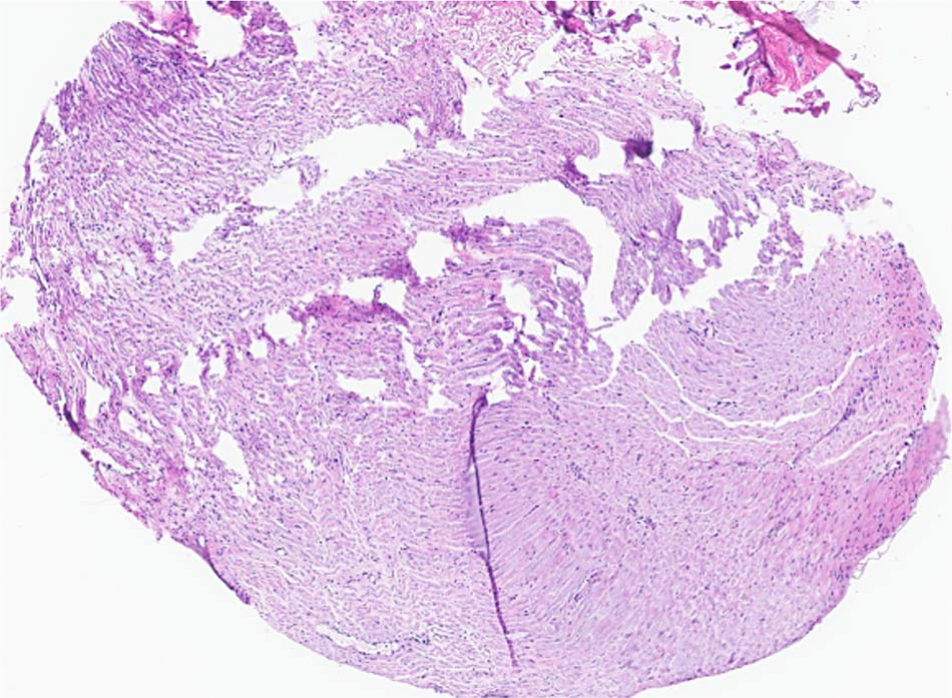
Facial nerve, left, proximal margin, biopsy: focally involved by invasive SCC.

**Figure 5:**
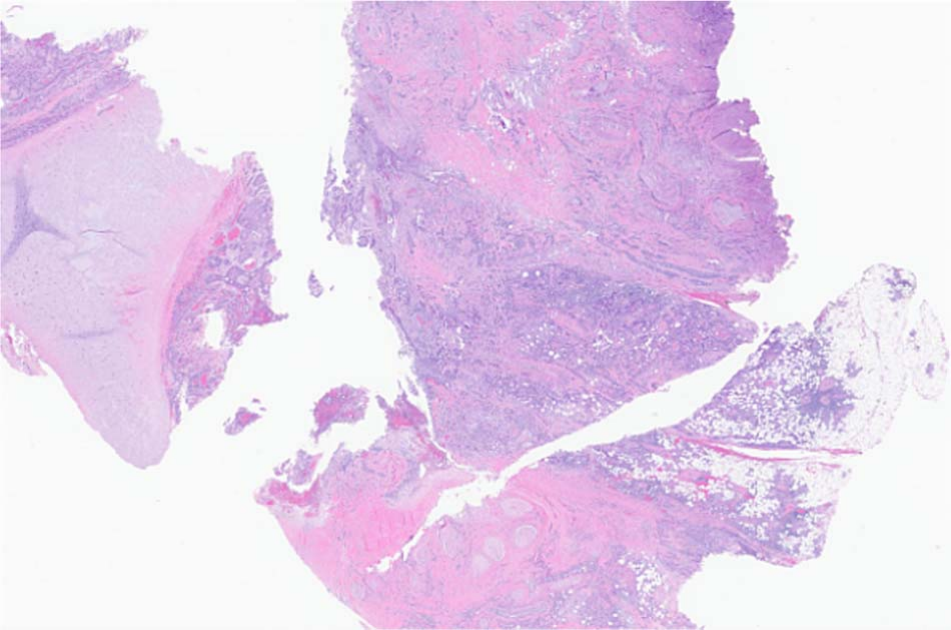
Ear, left, auriculectomy: invasive moderately differentiated SCC, forming a poorly demarcated infiltrating dominant mass, ~5.5 cm in size and 2 cm in depth of invasion; extensive nerve invasion and focal lymphovascular invasion.

Subsequent surgery included left partial auriculectomy, wide local excision, and lateral temporal bone resection. Pathology revealed lymphatic invasion (LVI), perineural invasion (PNI), and facial nerve and mastoid bone involvement (Figs 4 and 5). CLL-fluorescence in situ hybridization (FISH) revealed 13q deletion; Immunoglobulin Heavy Chain gene (IGH) somatic hypermutation analysis identified IGHV3–23*01 rearrangement (mutated level 5.7%). Due to prior radiation, six cycles of cemiplimab were planned. A follow-up PET-CT scan showed enlarged, FDG-avid cervical lymph nodes and soft tissue nodularity beneath the posterior margin of the graft.

**Figure 6:**
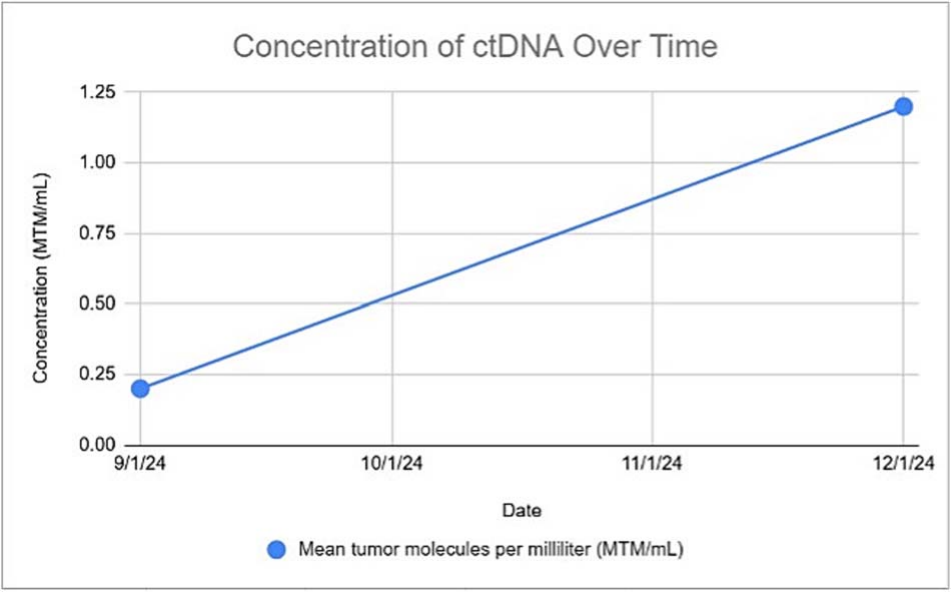
Concentration of ctDNA.

Genetic profiling revealed a high tumor mutation burden (50 mutations/Mb) with ARID1B, CDKN2A, MSH2, PMS2, and TP53 alterations. After 3 months, based on an uptrend in circulating DNA (ctDNA) levels (Signatera), cetuximab was initiated (Fig. 6*).* The biweekly regimen was continued, with follow-up PET scans conducted every 3 months. The PET scan conducted at 6 months confirmed a complete response.

## Discussion

This report highlights our experience with synchronous asymptomatic CLL and recurrent SCC involving the unilateral parotid gland. Immunosuppression, ear location, and histopathologic features, including tumor thickness, PNI, tumor differentiation and size, and desmoplastic growth, all increase the risk of SCC recurrence [7–9]. Current practice involves aggressive management of high-risk patients with radiation in addition to surgery or chemotherapy as appropriate. However, identification of high-risk patients without obvious signs, as may occur in asymptomatic CLL, remains a significant challenge.

Approximately 70% of CLL patients are asymptomatic at diagnosis. While treatment is not required treatment for asymptomatic CLL, [10] 30%–50% of these patients progress to symptomatic disease over time, with or without a change in staging. [11]. Hence, early identification of patients at risk for progression can enhance personalized management and risk reduction strategies.

Genetic profiling, utilizing FISH and immunoglobulin heavy-chain gene variable region (IgHV)=testing, common in other cutaneous cancers, serves as an effective risk stratification tool [12]. FISH detects genetic amplifications, translocations, and deletions. In our case, the absence of 17p and 11q deletions indicated no immediate treatment. Identification of a high TMB and DNA alteration provided a rationale for cemiplimab, while ctDNA monitoring enabled early detection of molecular progression and timely escalation to Cetuximab. Genetic testing, despite allowing for the recognition of progress before radiographic evidence emerges, remains underutilized due to systemic barriers [11]. In addition to progression, genetic profiling can also inform follow-up duration. A meta-analysis by Parikh *et al.* (2016) recommended FISH and IgHV as standard tests for newly diagnosed patients, though it didn’t specifically address asymptomatic patients [13]. We advocate for identifying high-risk patients in the early stage (RAI stage 0–1 or Binet stage A). This case report underscores the utility of FISH and IgHV testing in synchronous CLL and SCC. Additionally, we used ctDNA to monitor progression, supporting its role as a tumor burden biomarker [12].

## Conclusion

This case highlights the clinical utility of genetic profiling, including FISH, IgHV mutation analysis, and ctDNA surveillance, as a means of risk stratification in patients with synchronous malignancies such as SCC and asymptomatic CLL. While current guidelines do not mandate genomic testing in asymptomatic CLL or most salivary gland tumors, our experience highlights its value in uncovering clinically silent but prognostically significant disease that may influence recurrence risk, surveillance protocols, and treatment planning. In light of increasing evidence linking immunologic dysregulation in CLL with aggressive cutaneous malignancies, we advocate for a paradigm shift toward earlier and broader use of molecular diagnostics in complex or recurrent head and neck cancers, particularly when synchronous hematologic malignancies are present. Incorporating genomic risk stratification into routine evaluation may refine therapeutic decision-making and improve long-term oncologic outcomes.
